# Clinical application of Xianfang Huoming decoction in hidradenitis suppurativa: a three-case series and literature review

**DOI:** 10.3389/fmed.2026.1899685

**Published:** 2026-10-16

**Authors:** Zixi Wang, Xuling Wu, Hongliu Zhu, Ke Liu, Yihong Gao, Yuegang Wei, Qiuxia Mao, Liyan Zhou

**Affiliations:** 1 Department of Dermatology, Jiangyin Hospital of Traditional Chinese Medicine, Jiangyin Hospital Affiliated to Nanjing University of Chinese Medicine, Jiangyin, Jiangsu, China; 2 Department of Dermatology, Affiliated Hospital of Nanjing University of Chinese Medicine, Nanjing, Jiangsu, China

**Keywords:** case series, herbal medicine, hidradenitis suppurativa, traditional Chinese medicine, Xianfang Huoming decoction

## Abstract

Hidradenitis suppurativa (HS) is a chronic, recurrent inflammatory follicular disease characterized by painful nodules, abscesses, and sinus tract formation that typically affects intertriginous areas, such as the axillae, inguinal regions, and gluteal region. This condition is often persistent and is difficult to control in clinical practice. In recent years, traditional Chinese medicine has been explored as a potential adjunct in its management. Here, we summarize the cases of three male patients with HS treated at the Department of Dermatology, Jiangyin Affiliated Hospital of Nanjing University of Chinese Medicine (2023–2024), aged 12–29 years with disease durations of 6 months to 4 years. The lesions were primarily located in the axillae or gluteal region and presented as recurrent papules, pustules, nodules, and painful cysts. All cases were Hurley stage I–II and classified as heat-toxin accumulation syndrome in traditional Chinese medicine. All patients received individualized combination therapies incorporating Xianfang Huoming Decoction, along with topical treatments, antibiotics, and/or surgical intervention when indicated. After 1–5 weeks of treatment, the inflammatory lesions and pain symptoms improved, with IHS4 scores decreasing by approximately 71%–81%. Patient 1 subsequently experienced recurrence after treatment discontinuation and was lost to dermatological follow-up. These cases suggest that Xianfang Huoming Decoction may help reduce inflammatory activity and promote lesion resolution in HS, supporting its potential role as an adjunctive therapeutic option.

## 1 Introduction

Hidradenitis suppurativa (HS), historically known as acne inversa, is a chronic, recurrent, and inflammatory follicular disease that can be debilitating (1, 2). It mainly involves intertriginous areas rich in apocrine glands, such as the inguinal region (approximately 90%), axillae (69%), perianal and perineal regions (37%), and buttocks (27%) (3). Clinically, it is characterized by recurrent inflammatory nodules, abscesses, and sinus tract formation, with a chronic relapsing course that markedly impairs quality of life (4). The current management of HS includes topical and intralesional therapies, systemic medications, physical approaches, and surgery. Systemic treatments include antibiotics, retinoids, biological agents, corticosteroids, and immunosuppressants (5). However, these options are limited by insufficient topical efficacy, transient response to local injections, antibiotic resistance with long-term use, and adverse effects of retinoids, such as hepatotoxicity and teratogenicity. Biological agents are effective, but expensive (6, 7). Therefore, safe, effective, and accessible alternative or adjunctive therapies remain an unmet clinical need, and evidence for the use of traditional Chinese medicine (TCM) in HS remains limited.

Xianfang Huoming Decoction is a classical TCM formula composed of *Lonicera japonica* Thunb, *Paeonia veitchii* Lynch,*Angelica sinensis* (Oliv.)Diels, *Angelica dahurica* (Fisch. ex Hoffm). Benth. et Hook. f., *Fritillaria thunbergii* Miq., *Saposhnikovia divaricata* (Turcz.)Schischk., *Gleditsia sinensis* Lam., *Trichosanthes kirilowii* Maxim., *Boswellia carterii* Birdw., *Commiphora myrrha* Engl., *Citrus reticulata* Blanco, and *Glycyrrhiza uralensis* Fisch (8). According to TCM theory, the formula is traditionally used based on the principles of “clearing heat and detoxifying,” together with regulating qi, activating blood, and resolving masses, with the overall aim of alleviating suppurative inflammation. This retrospective case series describes the clinical application of Xianfang Huoming Decoction in patients with HS and provides preliminary clinical evidence regarding its potential therapeutic value.

## 2 Case presentation

### 2.1 Case 1

A 29-year-old male patient presented to the Dermatology Department of Jiangyin Affiliated Hospital of Nanjing University of Chinese Medicine on December, 2023, with a four-year history of recurrent bilateral axillary lesions. The lesions initially developed without an identifiable trigger, presented as scattered papules, pustules, nodules, and cyst-like lesions, and progressively increased in number over time. The patient experienced persistent pain and discomfort, and the lesions repeatedly recurred despite self-medication and multiple treatments received at other institutions.

Dermatological examination revealed multiple deep-seated tender inflammatory nodules, abscesses, and sinus tracts involving both axillae. Based on the presence of typical HS lesions (painful deep-seated inflammatory nodules, abscesses, and sinus tracts), characteristic anatomical distribution (bilateral axillary involvement), and a chronic recurrent course, the patient was diagnosed with HS (9), Hurley stage II. The baseline International Hidradenitis Suppurativa Severity Score System (IHS4) score was 14. IHS4 was calculated based on the number of inflammatory nodules (×1), abscesses (×2), and draining tunnels (×4), with scores classified as mild (≤3), moderate (4–10), and severe (≥11) disease (10).

According to the syndrome differentiation, the lesions were characterized by dark-red nodules and abscesses with sinus formation. The patient also reported heat intolerance, preference for sweet foods, and dry stool. Tongue examination revealed a light-red tongue with a thin yellow coating, and pulse examination demonstrated a wiry and rapid pulse. Based on these clinical manifestations and TCM findings, the condition was classified as a “heat-toxin accumulation” pattern. Laboratory examinations revealed elevated levels of liver enzymes (alanine aminotransferase 98 U/L, aspartate aminotransferase 90 U/L, and gamma-glutamyl transferase 109 U/L), hyperglycemia (fasting glucose 7.33 mmol/L, random glucose up to 8.5 mmol/L), elevated glycated hemoglobin (HbA1c 6.8%), and hypertriglyceridemia (4.59 mmol/L).

The patient was treated with Xianfang Huoming Decoction (composition: *Lonicera japonica* Thunb 9 g, *Paeonia veitchii* Lynch 6 g, *Angelica sinensis* (Oliv.)Diels 6 g, *Angelica dahurica* (Fisch. ex Hoffm.)Benth. et Hook. f. 3 g, *Fritillaria thunbergii* Miq. 6 g, *Saposhnikovia divaricata* (Turcz.)Schischk. 6 g, *Gleditsia sinensis* Lam. 6 g, *Trichosanthes kirilowii* Maxim. 6 g, *Boswellia carterii* Birdw. 6 g, *Commiphora myrrha* Engl. 6 g, *Citrus reticulata* Blanco 9 g, and *Glycyrrhiza uralensis* Fisch. 6 g. Herbal materials were purchased from the hospital pharmacy procurement system and underwent routine quality control procedures according to the hospital standards. The decoction was prepared using water extraction, yielding approximately 400 mL per dose, and was administered orally twice daily after meals in a warm state for one week. The patient also received oral roxithromycin (150 mg twice daily), topical fusidic acid (applied twice daily for one consecutive week), and underwent two sessions of surgical debridement.

One week after treatment initiation, a follow-up examination demonstrated marked flattening and darkening of the lesions with resolution of the indurated nodules and abscesses, although the drainage strip remained in place. The IHS4 score decreased from 14 at baseline (4 inflammatory nodules×1 + 3 abscesses×2 + 1 draining tunnel×4) to 4 after treatment (1 residual draining tunnel×4), representing a 71% reduction in the IHS4 score. The patient reported an improvement in pain symptoms. No adverse events were documented during the treatment period, and treatment tolerance was acceptable. The patient was scheduled for regular dermatological follow-ups; however, he did not return for subsequent visits as recommended. More than four months later, recurrence, defined as the development of new HS-related lesions, was reported through telephone follow-up, and the patient sought further treatment in the general surgery department in April 2024 (Figures 1A,B). No follow-up liver enzyme measurements were available because of the absence of a subsequent dermatological follow-up.

**Figure 1:**
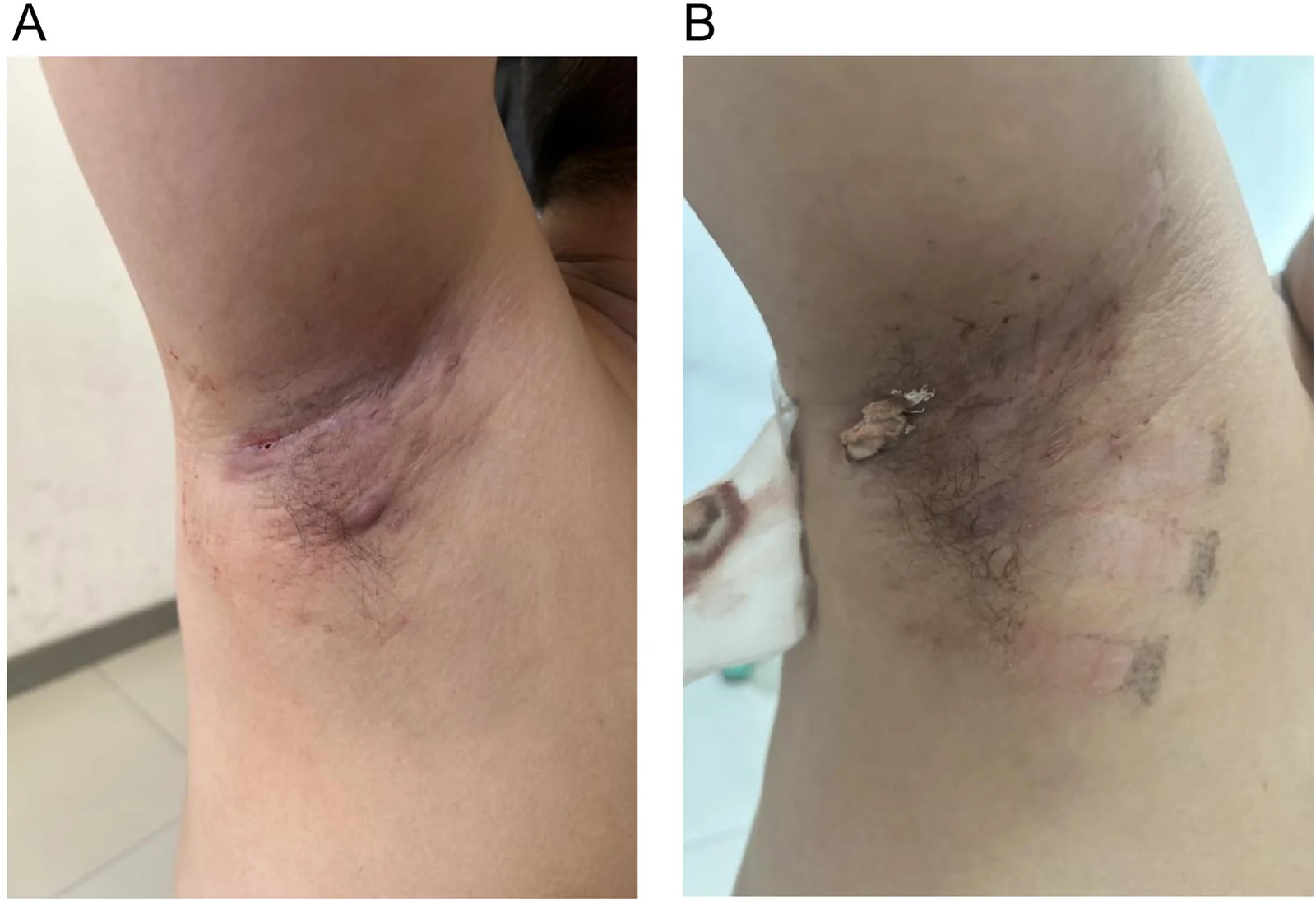
Clinical photographs of case 1 before and after treatment combined with surgical debridement. **(A)** Before treatment, showing multiple inflammatory nodules, abscesses, and draining sinus tracts in the bilateral axillary region. **(B)** One week after treatment, showing improvement of lesions with residual draining sinus tract. The lesions contributing to IHS4 scores were recorded according to the standard scoring system.

### 2.2 Case 2

A 12-year-old boy presented on June, 2023, with a two-year history of recurrent painful nodules on the buttocks. The number of lesions gradually increased with repeated relapses despite prior treatment. Physical examination revealed multiple deep-seated, tender nodules and abscesses without sinus tract formation. The baseline IHS4 score was 11 (9 inflammatory nodules  ×  1 + 1 abscess×2). Based on the presence of typical HS lesions (recurrent painful nodules and abscesses), typical anatomical distribution (buttock involvement), and a chronic recurrent disease course, the patient was diagnosed with HS (Hurley stage I) (9).

According to TCM syndrome differentiation, the patient presented with multiple nodules and abscesses on the buttocks, accompanied by marked tenderness on palpation, dry mouth, hyperhidrosis, and a habitual preference for cold beverages. Tongue examination revealed a light-red tongue with a thin white coating, and pulse examination revealed a rapid pulse. Based on the clinical manifestations in conjunction with the tongue and pulse findings, the condition was classified as the “heat-toxin accumulation” pattern.

The patient was treated with Xianfang Huoming Decoction (approximately 400 mL per dose, divided into two portions and taken orally after breakfast and dinner) for three weeks, combined with topical fusidic acid and clindamycin gel. During treatment, the patient was followed-up every two weeks, and topical antibiotics were continued as maintenance therapy after the initial treatment. At the first follow-up, most lesions had resolved, with only residual post-inflammatory hyperpigmentation, crusting, and one remaining abscess. The patient reported an improvement in pain symptoms. The IHS4 score decreased from 11 at baseline to 2 after treatment (calculated as 1 residual abscess×2), representing an 81% reduction in the IHS4 score. No adverse events were reported during treatment, and the regimen was generally well tolerated. The patient was subsequently followed-up by telephone for six months. During this period, no new HS-related lesions were reported and no disease recurrence was observed (Figures 2A,B).

**Figure 2:**
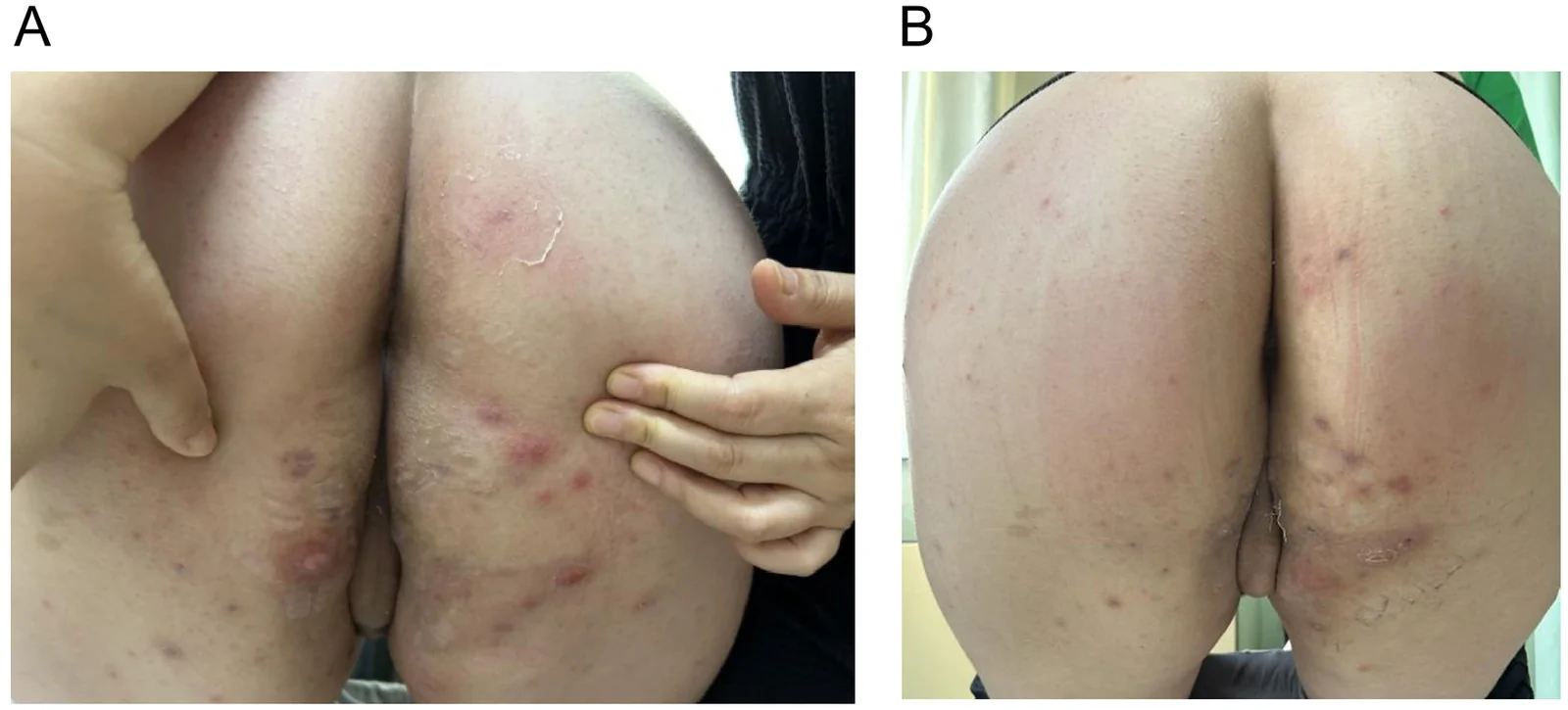
Clinical photographs of case 2 before and after 3 weeks of treatment. **(A)** Before treatment, showing multiple inflammatory nodules and abscesses in the buttock region. **(B)** After 3 weeks of treatment, showing marked improvement with one residual abscess. The lesions contributing to IHS4 scores were recorded according to the standard scoring system.

### 2.3 Case 3

A 14-year-old boy presented on July, 2024, with a six-month history of recurrent papules and pustules on the buttocks that persisted despite multiple prior treatments. Physical examination revealed multiple deep-seated, tender nodules and abscesses without sinus tract formation. The baseline IHS4 score was 7. Based on the presence of typical HS lesions (recurrent painful nodules and abscesses), typical anatomical distribution (buttock involvement) (9), and chronic recurrent disease course, the patient was diagnosed with HS (Hurley stage I). Additionally, the patient reported a positive family history of similar lesions. His father had experienced recurrent papules, pustules, nodules, cysts, and abscesses on the buttocks for many years and had undergone surgical treatment eight years previously.

According to the TCM syndrome differentiation, multiple erythematous papules were observed on the buttocks, accompanied by significant pain. The patient also reported chronic dry mouth without bitter taste, a preference for cold beverages, and a burning sensation in the perianal region. Urine was dark yellow, whereas bowel habits remained normal. Tongue examination revealed a light-red tongue with a thin, white, and greasy coating, whereas pulse examination demonstrated a floating and rapid pulse. Based on the clinical manifestations in conjunction with the tongue and pulse findings, the condition was differentiated as the “heat-toxin accumulation” pattern in TCM.

The patient was treated with Xianfang Huoming Decoction administered orally (approximately 400 mL per dose, divided into two portions and taken orally after breakfast and dinner) for five weeks. During the treatment period, the patient was followed-up every two weeks. At follow-up, most lesions had resolved, with only residual post-inflammatory hyperpigmentation and crusting, and one remaining abscess. The IHS4 score decreased from 7 at baseline (3 inflammatory nodules×1 + 2 abscesses×2) to 2 after treatment (1 abscess×2), representing a 71% reduction in the IHS4 score. No adverse events were reported during treatment, and the regimen was generally well tolerated. The patient was subsequently followed-up by telephone for six months. During this period, no new HS-related lesions were reported and no disease recurrence was observed (Figures 3A,B).

**Figure 3:**
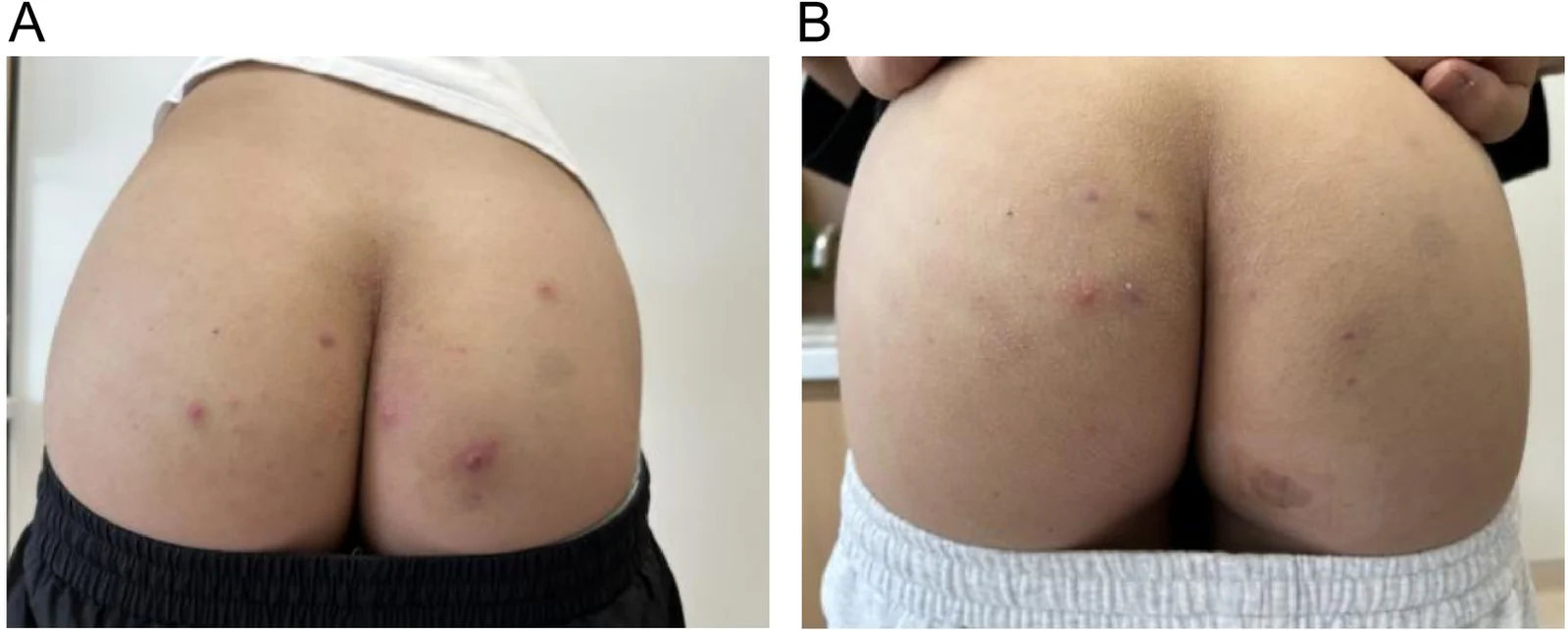
Clinical photographs of case 3 before and after 5 weeks of treatment. **(A)** Before treatment, showing inflammatory nodules and abscesses in the buttock region. **(B)** After 5 weeks of treatment, showing improvement with one residual abscess. The lesions contributing to IHS4 scores were recorded according to the standard scoring system.

## 3 Discussion

HS is a chronic inflammatory follicular disease characterized by recurrent, painful, deep-seated inflammatory lesions that may progress to abscesses, sinus tract formation, scarring, and contractures. Persistent ulcerative lesions may increase the risk of secondary squamous cell carcinoma (11, 12). The pathogenesis of HS is multifactorial, and involves genetic susceptibility, immune dysregulation, microbial imbalance, obesity, and smoking (13). These factors contribute to follicular hyperkeratinization, follicular occlusion, and rupture, followed by persistent perifollicular inflammation, tissue destruction, and recurrent disease (13). Clinically, HS typically involves the intertriginous and follicular-bearing areas, including the axillae, inframammary regions, groins, genital areas, and buttocks. The diagnosis of HS is primarily clinical and relies on three essential features: typical lesions, characteristic anatomical distribution, and chronic or recurrent disease course (14). Owing to its overlapping clinical manifestations, HS should be differentiated from other follicular inflammatory disorders. Recurrent furunculosis is mainly caused by deep bacterial infection of hair follicles, commonly associated with *Staphylococcus aureus*, and usually presents as acute tender erythematous nodules or abscesses rather than as chronic recurrent lesions in characteristic HS locations (15). Bacterial folliculitis is generally limited to superficial follicular inflammation, presenting as follicular papules and pustules with a relatively short course, and rarely shows the deep inflammatory nodules and chronic recurrence observed in HS (16). Pilonidal disease may also mimic gluteal HS, but is typically restricted to the midline natal cleft with sinus openings or hair entrapment, unlike the multifocal recurrent inflammatory lesions characteristic of HS (17).

Currently, international reports on the use of TCM for HS are limited. However, in China, many researchers are engaged in the clinical application of TCM for HS. A total of 27 studies on oral herbal treatment of HS are summarized (Table 1). Among these, 62.96% (17/27) were single-case reports and 29.63% (8/27) included more than 10 cases. Hypertriglyceridemia and elevated liver enzymes may also be associated manifestations or systemic components of HS. Previous studies have reported associations between elevated liver enzyme levels and systemic inflammation, disease severity, and metabolic syndrome in patients with HS, while a recent systematic review and meta-analysis found an association between lipid profiles and HS, further supporting the presence of metabolic abnormalities in this patient population (45, 46). Lesion distribution was most frequently reported in the perianal, anal, and perineal regions (55.55%, 15/27), followed by the buttocks (37.04%, 10/27), groin (14.81%, 4/27), and axillae (7.41%, 2/27). Regarding TCM syndromes, dampness was the most common (37.04%, 10/27), followed by heat (33.33%, 9/27), deficiency (29.63%, 8/27), and toxin patterns (22.22%, 6/27). In terms of Hurley staging, 55.55% (15/27) of cases were stage III. Disease duration was generally long term (years), with only two reports describing disease duration within 1 year. Therapeutically, 74.07% (20/27) combined surgery, and 62.96% (17/27) combined antibiotics, with only one study using TCM monotherapy. Several authors have proposed syndrome-based treatment strategies. Ma et al. (21), Li K (23), and Hu et al. (44) emphasized individualized treatment according to local lesions and systemic conditions with dynamic modification of herbal prescriptions during disease progression. Some studies also proposed TCM pathogenesis models based on accumulated clinical experience. Wang (22) attributed perianal HS to damp-heat accumulation in the liver meridian with toxin stagnation and treated it with Longdan Xiegan Decoction to clear damp-heat and detoxify it, followed by long-term administration for constitutional regulation. Li L (25) identified two main patterns: damp-toxin accumulation treated with modified Wuwei Xiaodu Yin, and deficiency with pathogen invasion treated with modified Tuoli Xiaodu San to strengthen healthy qi and resolve toxins. Fan (47) proposed a model of congenital spleen dampness and liver hyperactivity with acquired dietary and lifestyle imbalances, leading to long-term accumulation of dampness, heat, and toxins. Two major TCM patterns were identified: heat-toxin accumulation, treated with clearing heat and detoxifying prescriptions (e.g., Wuwei Xiaodu Yin combined with Xianfang Huoming Decoction), and stasis-toxin binding, treated by activating blood circulation and resolving masses (e.g., Taohong Siwu Decoction combined with Xianfang Huoming Decoction).

**Table 1:** Review of cases on the treatment of acne inversa with traditional Chinese medicine.

Author(s)	Year of publication	Number of cases	Affected site	Syndrome pattern	Classification	Disease duration	Commonly used prescriptions or therapeutic principles	Combination therapy
He Z (18)	1990	1 case	Perianal region and buttocks	Damp-heat pouring downward	Hurley stage III	3 years	Clearing heat and cooling the blood; activating blood and resolving toxicity; dispelling dampness and unblocking the channels	Incision and drainage + herbal fumigation and washing
Long Q (19)	1997	1 case	Buttocks	Not specified	Hurley stage III	6 years	Modified combination of *Sijin’gang* and *Wuhu Xiaxichuan* formulas	Topical antibiotics
Luo X (20)	1998	15 cases	Perianal region	Not specified	Hurley stage II–III	3 months–24 years	Compound Qinbai Granules	Surgery
Ma Y (21)	1998	1 case	Left buttock	Internally generated damp-heat	Hurley stage III	>10 years	Modified *Wuwei Xiaodu Decoction* combined with *Tuonong Decoction* → *Bazhen Decoction*	Surgery; antibiotics
Wang D (22)	2000	22 cases	Perianal region	Damp-heat accumulation in the Lung meridian	Hurley stage I–III	Not specified	Modified *Longdan Xiegan Decoction*	Surgery
Li K (23)	2000	1 case	Axilla, vulva, groin	Qi and Yin deficiency with binding of damp toxin	Hurley stage II	14 years	Eliminating dampness and resolving toxicity → tonifying Qi and nourishing Yin → *Buzhong Yiqi Pills* + *Wuwei Xiaodu Pills* (concentrated)	None
Tang X (24)	2001	51 cases	Anal canal	Not specified	Hurley stage II–III	3 months–12 years	Heat-clearing, dampness-draining, and blood-cooling formulas	Surgery; antibiotics
Li L (25)	2005	72 cases	Perianal region	Deficiency with exuberant phlegm-dampness and accumulation of damp toxin	Hurley stage I	1–9 years	Modified *Wuwei Xiaodu Decoction* and *Tuoli Xiaodu Powder*	Surgery
Hong Z, et al. (26)	2012	2 cases	Not specified	Heat toxin in the Lung meridian	Not specified	Not specified	Treatment based on syndrome differentiation focusing on clearing heat, resolving toxicity, and cooling blood	Surgery; antibiotics
Li L, et al. (27)	2012	1 case	Vulva	Not specified	Hurley stage II	5 years	Harmonizing the nutritive level and expelling toxin; dissipating masses and reducing swelling	Antibiotics
Lu H, et al. (28)	2012	1 case	Perianal region	Deficiency in root and excess in branch	Hurley stage II	3 years	Modified *Tuoli Xiaodu Powder*	Surgery
Li Y, et al. (29)	2013	1 case	Perianal region	Damp-heat pouring downward	Hurley stage III	>20 years	Modified *Longdan Xiegan Decoction*	Incision and drainage
Wei W, et al. (30)	2013	1 case	Groin and perineum	Deficiency in root and excess in branch; mixed deficiency and excess	Hurley stage III	7 years	Modified *Tuoli Xiaodu Powder* combined with *Longdan Xiegan Decoction*	Surgery
Zhao Y, et al. (31)	2014	1 case	Groin, perianal region	Deficiency in root and excess in branch	Hurley stage III	7 years	Modified *Longdan Xiegan Decoction* combined with *Tuoli Xiaodu Powder*	Surgery; antibiotics
Wang H (32)	2015	10 cases	Perianal region	Not specified	Hurley stage III	1–15 years (mean 7 years)	*Xianfang Huoming Decoction*, *Bixie Shenshi Decoction*, *Guipi Decoction*, or self-formulated prescriptions	Surgery; antibiotics
Zhao L (33)	2016	25 cases	Perianal region	Damp-heat or phlegm-dampness	Hurley stage III	2–7 years (mean 3.1 ± 0.2 years)	*Longdan Xiegan Decoction*	Surgery; antibiotics
Zeng J, et al. (34)	2016	4 cases	Perianal region	Not specified	Hurley stage III	9 months–2 years	Modified *Longdan Xiegan Decoction*	Surgery; antibiotics; retinoids
Hou J, et al. (35)	2016	1 case	Buttocks	Not specified	Hurley stage III	>10 years	Heat-clearing and detoxifying herbal decoction for reducing swelling and resolving induration	Surgery; red light therapy; antibiotics
Zhang S (36)	2016	1 case	Perianal region, buttocks, perineum	Heart and Spleen deficiency	Hurley stage III	>10 years	Modified *Jianpi Pills*	Surgery; antibiotics
Zhou K, et al. (37)	2017	1 case	Head, face, posterior neck, axilla, groin	Internal exuberance of phlegm-dampness	Hurley stage I	5 years	Institutional preparations (*Piyan No.3 Granules*, etc.)	Antibiotics; corticosteroids
Liu Y (38)	2017	10 cases	Not specified	Not specified	Not specified	Not specified	Acne Granules	Photodynamic therapy; fire needle
Xu X, et al. (39)	2018	1 case	Buttocks	Accumulation of phlegm-dampness and heat toxin	Hurley stage II	>1 year	Modified *Wuwei Xiaodu Decoction* combined with *Sanren Decoction*	Surgery; antibiotics
Gao J, et al. (40)	2019	15 cases	Perianal region	Intense heat toxin; deficiency heat	Hurley stage III	6–56 months (mean 24.3 ± 18.3 months)	Self-formulated heat-clearing and dampness-draining decoctions	Surgery; antibiotics
Long J, et al. (41)	2019	1 case	Right buttock	Damp-heat accumulation pattern	Hurley stage III	17 years	Modified *Xianfang Huoming Decoction* combined with *Wuwei Xiaodu Decoction*	Surgery; antibiotics
Xie Q, et al. (42)	2021	1 case	Buttocks, head, back, axilla	Dysfunction of Xuanfu opening and closing	Hurley stage III	6 years	Wind-dispersing Xuanfu-regulating medicinals	Surgery
Liu L, et al. (43)	2022	1 case	Head/face, left axilla, right buttock, scrotum	Dysfunction of Spleen and Stomach	Hurley stage II	10 years	Qingfu No.3 preparation	Surgery; antibiotics
Hu Y, et al. (44)	2024	1 case	Bilateral buttocks, perineum, thighs	Deficiency of vital Qi with exuberant toxin	Hurley stage III	>10 years	Modified *Tuoli Xiaodu Powder* → *Lizhong Decoction*, *Guizhi Decoction*, *Tuonong Powder* → Jianpi Yangxue paste	Surgery; antibiotics

In TCM theory, Xianfang Huoming Decoction is commonly used for suppurative inflammatory disorders characterized by swelling, pain, and abscess formation. In the present study, Xianfang Huoming Decoction was included as a major component of the therapeutic regimen, with other treatments selected according to individual clinical conditions. Case 1, the most severe of the three, had a 4-year disease history and Hurley stage II disease. It received Xianfang Huoming Decoction combined with incision and drainage and antibiotics, achieving a rapid response within 1 week, with the IHS4 score decreasing from 14 to 4 (71% reduction). However, recurrence occurred more than 4 months after discontinuation, likely due to an insufficient treatment course, highlighting the importance of continuing medication after symptom resolution to consolidate the therapeutic effects. In addition, abnormal liver function and hyperglycemia may have contributed to rapid relapse, suggesting that endocrine and metabolic abnormalities should be considered in young patients with HS and that combined management may improve outcomes and reduce recurrence. In the reviewed literature, among patients not treated with surgery, three Hurley stage III patients received combinations of herbal medicine with incision and drainage, fumigation therapy, or topical antibiotics, and two Hurley stage II patients were treated with either herbal monotherapy or herbal medicine combined with oral antibiotics. Compared with conventional therapies, the potential role of Xianfang Huoming Decoction as an adjunctive approach requires further evaluation in comparative stage-stratified studies. Although clinical improvements were observed within a relatively short treatment period, the optimal treatment duration and comparative effectiveness require further investigation.

Recent studies have also highlighted the involvement of dysregulated innate and adaptive immune responses in the pathogenesis of HS (48). Key inflammatory pathways include the activation of TNF-α, IL-1, IL-17, and IL-23 signaling, which contribute to sustained inflammation, tissue destruction, and disease progression (49). Systemic inflammatory markers such as C-reactive protein and erythrocyte sedimentation rate have been investigated as potential indicators of disease activity and severity (50, 51). In the present case series, inflammatory biomarkers were not evaluated; therefore, the potential biological mechanisms underlying the observed clinical improvement following treatment regimens incorporating Xianfang Huoming Decoction remain speculative and require further investigation.

Overall, these cases describe the clinical improvement observed in patients with HS receiving a multimodal therapeutic approach incorporating Xianfang Huoming Decoction and conventional treatments. These findings provide preliminary observations regarding its potential role in HS management. However, its independent efficacy and long-term effects require further validation.

## 4 Limitation and conclusion

This case series has several limitations. First, pain improvement was evaluated based on patients’ subjective reports rather than on standardized pain assessment tools, which may have introduced a potential reporting bias. Second, Case 1 presented with elevated liver enzyme levels before treatment; however, the patient did not return for the scheduled follow-up after subsequent treatment; therefore, follow-up evaluation of liver function was unavailable. This limited the assessment of the safety profile of the combined therapeutic approach. In addition, the small sample size, heterogeneous clinical presentations, concomitant conventional treatments, and absence of a control group prevented determination of the independent efficacy and safety of Xianfang Huoming Decoction. Furthermore, the concentration of active compounds in herbal remedies may vary according to the season of collection, intra-species variation, weather, and growing conditions, which may pose challenges to the consistency and standardization of herbal preparations. The potential interactions between herbal remedies and concomitant medications, such as oral antibiotics, should also be considered and further investigated.

This case series describes the clinical improvement in three patients with hidradenitis suppurativa treated with Xianfang Huoming Decoction, either alone or in combination with conventional therapies. Given the concomitant treatments, small sample size, heterogeneous clinical presentations, and absence of a control group, the independent efficacy and safety of herbal formulations cannot be established. Prospective controlled studies using standardized clinical outcomes, safety assessments, and objective inflammatory biomarkers are warranted.

## Data Availability

The original contributions presented in the study are included in the article/Supplementary Material, further inquiries can be directed to the corresponding authors.
